# Design and Characterization of Glyceryl Monooleate-Nanostructures Containing Doxorubicin Hydrochloride

**DOI:** 10.3390/pharmaceutics12111017

**Published:** 2020-10-24

**Authors:** Agnese Gagliardi, Donato Cosco, Betty P. Udongo, Luciana Dini, Giuseppe Viglietto, Donatella Paolino

**Affiliations:** 1 Department of Experimental and Clinical Medicine, University “Magna Græcia” of Catanzaro, Campus Universitario “S. Venuta”, I-88100 Catanzaro, Italy; gagliardi@unicz.it (A.G.); Viglietto@unicz.it (G.V.); 2 Department of Health Sciences, University “Magna Græcia” of Catanzaro, Campus Universitario “S. Venuta”, I-88100 Catanzaro, Italy; donatocosco@unicz.it; 3 Pincer Training and Research Institute, Plot 1127, Lukuli Zone 5 00256, Uganda; bettyudongo@gmail.com; 4 Department of Biology and Biotechnologies Charles Darwin, Sapienza University of Rome, 00185 Rome, Italy; luciana.dini@uniroma1.it

**Keywords:** glyceryl monooleate, nanostructures, doxorubicin hydrochloride, poloxamers, polisorbates, multidrug resistance

## Abstract

Glyceryl monooleate (GMO) is one of the most popular amphiphilic lipids, which, in the presence of different amounts of water and a proper amount of stabilizer, can promote the development of well defined, thermodynamically stable nanostructures, called lyotropic liquid crystal dispersions. The aim of this study is based on the design, characterization, and evaluation of the cytotoxicity of lyotropic liquid crystal nanostructures containing a model anticancer drug such as doxorubicin hydrochloride. The drug is efficiently retained by the GMO nanosystems by a remote loading approach. The nanostructures prepared with different non-ionic surfactants (poloxamers and polysorbates) are characterized by different physico-chemical features as a function of several parameters, i.e., serum stability, temperature, and different pH values, as well as the amount of cryoprotectants used to obtain suitable freeze-dried systems. The nanostructures prepared with poloxamer 407 used as a stabilizer show an increased toxicity of the entrapped drug on breast cancer cell lines (MCF-7 and MDA-MB-231) due to their ability to sensitize multidrug-resistant (MDR) tumor cells through the inhibition of specific drug efflux transporters. Moreover, the interaction between the nanostructures and the cells occurs after just a few hours, evidencing a huge cellular uptake of the nanosystems.

## 1. Introduction

Glyceryl monooleate (GMO) is often described as a special lipid that plays an important role in drug delivery systems, due to its ability to self-assemble in water and to form a variety of well-defined, thermodynamically stable liquid crystal structures [1]. It also exhibits long-range order in one, two, or three dimensions [2,3]. GMO is widely known as a non-toxic, biodegradable and biocompatible product. It received generally recognized as safe (GRAS) status from the Food and Drug Administration (FDA) and is included in the Inactive Ingredients Guide [4,5].

In the presence of a proper stabilizer [6], liquid crystal phases can be arranged in different supramolecular structures such as cubosomes [7,8] or hexosomes [9,10,11], which could potentially be used for intravenous injection. This is because of their scarce viscosity as well as their ability to maintain the internal nanostructure of the bulk systems and keep it intact. Moreover, their stability in aqueous environments, their inexpensive raw materials as well as their well-defined structure and their higher membrane surface area allow them to retain significantly larger amounts of drugs then their lamellar counterparts such as liposomes [12]. The excellent characteristics of these hosting matrices make these nanostructures excellent and promising carriers for various applications in the drug delivery field [13].

The addition of surfactants can indeed influence the behavior of lipids in phases and are an important factor in preventing destabilizing phenomena of the colloidal dispersions in aqueous media, thus improving their shelf-life [3]. Among the stabilizing agents, the most extensively employed for the preparation of the lyotropic liquid crystalline nanostructures are poly-oxy-ethylene(PEO)-based surfactants such as poloxamers and polysorbates. In particular, poloxamers are nonionic triblock copolymers composed of a central hydrophobic portion of polyoxypropylene oxide (PPO) linked to two hydrophilic blocks of poly-ethylene oxide (POE) [14,15]. Their peculiar characteristics depend on the lengths of the chains of the various units, and on the different molecular weights and physical forms. Indeed, they act as steric stabilizers by way of the adsorption of the hydrophobic units onto the surfaces of nanostructures, which prevents the fusion of particles and thus promotes the physical stability of a formulation [16,17].

Moreover, polysorbates (PS) are nonionic, hydrophilic stabilizers made up of fatty acid esters of polyoxyethylene sorbitan [18]. Their bone structure consists of a sorbitan ring with ethylene oxide polymers linked at three different hydroxyl positions. Their molecular weight is significantly lower, and they have shorter hydrophilic PEO chains, in addition to a hydrophobic anchor consisting of fatty acid. The current study was designed to compare GMO-nanostructures prepared with the most popular classes of non-ionic surfactants (polysorbates and poloxamers) as a function of temperature, serum stability at various pH values, and the cryoprotectants used to obtain suitable freeze-dried systems. The most promising formulations were chosen to encapsulate the hydrophilic anticancer drug, doxorubicin hydrochloride (DOX).

DOX is one of the widely used powerful antineoplastic agents in clinical practice for the treatment of various cancers, hematological malignancies, soft tissue sarcomas, and solid tumors [19]. In order to increase its solubility in water, it is generally used in its hydrochloride form [20]. Unfortunately, the use of DOX revealed significant tissue toxicities, such as acute and chronic cardiotoxicity, which frequently contribute to fatal congestive heart failure. In addition, the intrinsic multidrug resistance of some cancer cells along with the poor penetration and distribution in the solid tumor tissues of DOX resulted in the failure of its pharmacological efficacy.

Several GMO-based nanosystems used for the delivery of DOX [21,22,23,24] have been widely implemented and described in literature but in this experimental work, for the first time (to the best of our knowledge), a remote loading approach was used to enhance the entrapment efficiency of the drug, which is normally employed to achieve a high retention rate in liposomes [25]. The cytotoxicity of the nanosystems was also investigated on different breast cancer cell lines (MCF-7 and MDA-MB-231). Finally, the degree of interaction between the GMO-nanostructures and the cells was evaluated by means of radiolabelled markers (such as tritiated hexadecyl-cholesterol) and confocal laser microscopy (CLSM).

## 2. Materials and Methods

### 2.1. Materials

Glyceryl monooleate (Monomuls 90.018) with purity >90%, 3-(4,5-dimethylthiazol-2-yl)-3,5-diphenyltetrazolium bromide salt (used for MTT-tests), doxorubicin hydrochloride, phosphate buffered saline (PBS) tablets, dimethyl sulfoxide, amphothericin B solution (250 μg/mL), rhodamine-1,2-dihexadecanoyl-*sn*-glycero-3-phosphoethanolamine triethylammonium salt (rhodamine-DHPE) were purchased from Sigma Aldrich (Milan, Italy) (for brevity, poloxamers are indicated in the main text using their trade name Pluronic^®^ with BASF-assigned identifying code, while the polysorbates are indicated with their trade name Tween^®^). Poloxamer 407 (Pluronic F127, PL F127), poloxamer 188 (Pluronic F-68, PL F68) and Pluronic 10,500 were purchased from BASF (Ludwigshafen, Germany). Tween 80 (T80), Tween 60 (T60), Tween 40 (T40), Tween 20 (T20) were provided by Acef S.p.a. (Piacenza, Italy) Ethanol was obtained from Carlo Erba SpA (Rodano, Italy), while cellulose membrane Spectra/Por MWCO 10 kDA was obtained from Spectrum Laboratories Inc. (Eindhoven, the Netherlands).

For the in vitro studies, Dulbecco’s modified Eagle’s Medium (DMEM) enriched with Glutamax I, tripsin/EDTA, penicillin/streptomycin solution and fetal bovine serum (FBS) were obtained from Gibco (Thermo Fisher Scientific, Waltham, MA, USA). MCF-7 and MDA-MB-231 cells were purchased from the IRCCS Azienda Ospedaliera Universitaria San Martino-IST Istituto Nazionale per la Ricerca sul Cancro (Genova, Liguria). [^3^H] cholesteryl hexadecyl ether ([^3^H]CHE, 40 Ci/mmoL) was obtained from Perkin Elmer-Italia (Monza, Italy).

### 2.2. Preparation of GMO-Nanostructures

The GMO-nanostructures were prepared by emulsifying a GMO-ethanol solution with an aqueous phase containing non-ionic surfactants under a high energy process. In detail, 90 mg of Monomuls 90.018 (GMO) was completely dissolved in ethanol (4 mL), while the surfactant solution (poloxamers/polysorbates) (0.4% *w*/*v*) was prepared in milliQ water (10 mL). The aqueous solution was added drop-by-drop to the organic phase, under continuous stirring, using an Ultraturrax T25 basic homogenizer (IKA^®^-Werke GmbH & Co. KG, Staufen, Germany), at a mixing speed of 18,500 rpm (3 different cycles of 5 min) at room temperature as previously reported [6]. The nanostructures containing DOX were obtained by adding different amounts of the drug to the aqueous phase. In addition, a remote loading method was used to improve drug entrapment. The aqueous phase was first hydrated with ammonium sulfate solution (250 mM). The resulting nanostructures were centrifuged by Amicon^®^ Ultra centrifugal filters (Sigma Aldrich Co, St Louis, MO, USA) (4000 rpm for 120 min) in order to remove the unentrapped ammonium sulphate solution. The pelletized vesicles were resuspended with a DOX solution (1 mg/mL). The samples were then incubated for 3 h and under continuous gentle stirring in order to facilitate the encapsulation of the DOX in the vesicles. The DOX-loaded nanostructures were centrifuged again in order to remove the non-encapsulated drug and finally resuspended with aqueous solution just before using.

### 2.3. Physico-Chemical Characterization

Mean size, size distribution and zeta potential were investigated using photon correlation spectroscopy (PCS) (Zetasizer Nano ZS, Malvern Panalytical Ltd., Spectris plc, Worchestershire, England) as previously described [26]. The results were expressed as the mean of three different experiments ± standard deviation.

For cryo-TEM imaging, liquid samples (10 μL) were dropped on a lacey carbon film-covered copper grid (TAAB) (Laboratories Equipment Ltd, Calleva Park Aldermaston, Berks, RG7 8NA, England), the excess sample was removed with filter paper and then the grid was quickly dipped in liquid nitrogen using a cryogenic chamber. The grid thus prepared can either be stored in liquid nitrogen before observation using electron microscopy or directly observed. Cryo-TEM observations were performed using a Hitachi 7700 microscope (Hitachi, Tokyo, Japan) and images were processed by EMIP Image Processing Software (Hitachi High Technologies, Corporation, Tokyo, Japan)).

The stability of the GMO-nanostructures was evaluated using a Turbiscan Lab^®^ Expert (Formulaction, Toulouse, France) apparatus following the variation of their backscattering (ΔBS) profiles as a function of time. The data were processed using a Turby Soft 2.0 (Formulaction, Toulouse, France) and expressed as Turbiscan stability index (TSI) versus time [27,28]. The TSI values were calculated using Equation (1) with a specific computer program:

(1) $$\mathrm{TSI}=\sqrt{\frac{\sum_{i=1}^{n}{\left(x_{i}-x_{\mathrm{BS}}\right)}^{2}}{n-1}}$$

where x_i_ is the backscattering for each minute of measurement, x_BS_ is the mean x_i_, and n is the number of scans [29].

#### 2.3.1. Influence of Temperature, pH and Serum on the Stability of Nanostructures

The influence of temperature on the physico-chemical properties of various GMO formulations was investigated by incubating the samples in a water bath for 1 h at fixed temperatures ranging from 30 to 50 °C, as previously described [30].

Moreover, the behavior of the formulations was studied by dispersing the samples (dilution 1:50) in deionized water at different pH values (4.0, 6.0, 8.0, 10.0, by a pH meter Seven Compact S210 Mettler Toledo, Columbus, OH, USA), using 1 mol/L NaOH or HCl, for 1 h under slow, continuous stirring at room temperature. In addition, the nanostructures were incubated in 70% FBS [31]. Briefly, 200 μL samples of the formulations were added to 1 mL FBS and incubated at 37 °C for 48 h and analyzed at different incubation times (0,5, 1, 2, 3, 4, 6, 24 and 48 h), as previously described [32].

#### 2.3.2. Freeze-Drying of Nanosystems

Lyophilization of the GMO nanostructures was performed by using a freeze-drying system (VirTis SP scientific sentry 2.0; SP Industries, Warminster, PA, USA) equipped with a vacuum pump (B14 model; Carpanelli S.p.a., Bologna, Italy) as previously reported [30]. In detail, several cryoprotectants (namely glucose, trehalose, sucrose, mannose, mannitol) at various concentrations (5 and 10% *w*/*v*) were added to the samples in Pyrex glass vials, and then frozen in liquid nitrogen for 2 min. The nanosystems were then placed in the freeze-drying chamber (VirTis SP scientific sentry 2.0; SP Industries, Warminster, PA, USA) and subjected to cryo-drying for 24 h. Subsequently, the resulting powder was rehydrated with the same volume of sublimated water, manually shaken and then the mean size and zeta potential were evaluated as previously reported.

### 2.4. Entrapment Efficiency of DOX

The entrapment efficiency of doxorubicin was evaluated by means of suitable spectrophotometric analysis. The GMO-formulations prepared with various amounts of drugs (0.1–0.6 mg/mL) were centrifuged using Amicon^®^ Ultra centrifugal filters (4000 rpm for 60 min). The amount of drug contained in the solutions was spectrophotometrically determined (Lambda 35; Perkin Elmer, Waltham, MA, USA) at λ max 480 nm. No interference deriving from the empty formulation was observed. The amount of drug entrapped in the nanostructures was determined as the difference between the amount added during the preparation of the nanosystems and the non-encapsulated amount that remained. The entrapment efficiency (EE%) was expressed as the percentage of the total amount of drug that became entrapped, according to the following Equation:

EE% = D_E_/D_T_ × 100 (2)

where D_E_ is the total amount of compound added to the formulation during the preparation procedure, and D_T_ is the amount of unentrapped drug obtained after the purification procedure.

In addition, the loading capacity (LC %) was calculated as the percentage of the ratio between the amount of total entrapped DOX and the total weight of the nanostructures.

### 2.5. DOX Release from GMO-Nanostructures

The amount of doxorubicin released from the GMO-nanostructures was evaluated by means of the dialysis method using cellulose acetate dialysis tubing (Spectra/Por with molecular cutoff 12,000–14,000 by Spectrum Laboratories Inc., Eindhoven, The Netherlands) sealed at both ends with clips [33]. A PBS solution (pH 7.4, 0.1 M) constantly stirred and warmed to 37 ± 0.1 °C was used as the release fluid for the active compound. The drug-loaded nanosystems (1 mL) were placed into dialysis bags (Spectrum Laboratories Inc., Eindhoven, The Netherlands), which were then transferred into beakers containing 200 mL of the release medium. At predetermined time intervals, 1 mL of the release medium was withdrawn and replaced with fresh. Samples were then analyzed by spectrophotometric analysis. The percentage of released DOX, before and after pretreatment with ammonium sulfate, was calculated using the following Equation:

Release (%) = drug_rel_/drug_load_ × 100 (3)

where drug_rel_ is the amount of drug released at the time t and drug_load_ is the amount of drug entrapped within the GMO nanostructures.

### 2.6. Cell Cultures and In Vitro Cytotoxicity

MCF-7 (breast cancer cell lines) and MDA-MB-231 cells (human breast adenocarcinoma) were incubated in plastic culture dishes (100 mm × 20 mm) in a water-jacketed CO_2_ incubator (Thermo Scientific, Dreieich, Germany) at 37 °C (5% CO_2_) using a DMEM with glutamine, supplemented with penicillin (100 UI/mL), streptomycin (100 ug/mL), amphotericin B (250 ug/mL) and FBS (10%, *v*/*v*), as previously described [34]. MTT-assay was used to evaluate cell viability after incubation with DOX in the free form (solubilized in water) or encapsulated in GMO-nanostructures and the investigation was performed as a function of the drug concentrations (0.01–5 μM) and incubation times (24, 48 and 72 h). The untreated cells were used as control while the cells treated with empty nanosystems were used as the blank. Successively, 20 μL of tetrazolium salt solubilized in phosphate buffered solution (5 mg/mL) was added to each well, the plates were incubated again for 3 h and then analyzed using a microplate spectrophotometer (xMARK™BIORAD, Bio-Rad Laboratories Inc., Hercules, CA, USA) at a wavelength of 540 nm with reference at 690 nm. Cell viability, expressed as a percentage, was reported as the mean of five different experiments ± standard deviation and was obtained through the following Equation, in which AbsT is the absorbance of treated cells and AbsC is the absorbance of control (untreated) cells:

Cell viability (%) = AbsT/AbsC × 100(4)

Moreover, the cell viability was further evaluated by trypan blue dye exclusion assay, as previously described. [35] In detail, cells exposed to compounds were trypsinized, the pellet was re-suspended in 0.4% trypan blue buffer, and cells were counted in a Neubauer chamber (Sigma Aldrich Co, St Louis, MO, USA). Cell death was expressed as the percentage of stained cells over total cells, which were counted.

#### Cytotoxicity of DOX-Loaded Nanostructures with Verapamil

MCF-7 and MDA-MB-231 cells were pretreated with verapamil, a first-generation multidrug-resistant (MDR) efflux pump inhibitor, before the incubation of the various DOX formulations in order to assess the reversal of MDR by P-glycoprotein (Pgp) inhibitors [36]. Briefly, the cancer cells were pretreated with 20 µM verapamil for 2 h and then treated with an increasing concentration of doxorubicin (0.01–5 μM) as a function of incubation times (24, 48 and 72 h) and then cytotoxicity was measured by way of MTT assay [37].

### 2.7. Interaction of Tritiated GMO-Nanostructures with Cells

The rate of interaction between the GMO-nanostructures and the cell lines (MCF-7 and MDA-MB-231) was investigated as a function of incubation times as previously described [30]. Briefly, tritiated nanostructures ([^3^H]CHE-radiolabeled systems, 0.003% *w*/*w* with respect to the amount of GMO) were prepared, and before each analysis they were centrifuged (80,000× *g* for 30 min at 4 °C) and resuspended in a suitable medium to avoid the interference of the [^3^H]CHE molecules that were not integrated into the structures of the nanosystems.

### 2.8. Cell Interaction by CLSM

The interaction between the cells and the nanostructures was investigated by confocal laser scanning microscopy (CLSM) (Leica Microsystems, Wetzlar, Germany), as previously described [27]. In detail, 4 × 10^4^ cells/mL were inserted in 6-well culture plates with a sterile glass slide on the bottom, incubated for 24 h, treated with nanostructures containing rhodamine-DHPE (0.1% molar) and then incubated again as a function of time. Successively, each well was washed with PBS three times and then the cells were fixed on the sterile glass slides with 1 mL of an ethanolic solution (70% *v*/*v*). After washing each slide glass once more with PBS (3 times in all), the cell nuclei were stained with a Hoechst solution (0.01 µg/mL). In order to remove enclosed air, cover-glasses were located on slides by means of a glycerol solution (70% *v*/*v*) and fixed using a transparent glue. A Leica TCS SP2 MP CLSM apparatus (Leica Microsystems, Wetzlar, Germany) was used to perform the analysis at λexc = 560 nm e λem = 580 nm for rhodamine probe and λexc = 405 nm and λem = 460 nm for Hoechst. A scan resolution of 1024 × 1024 pixels was used.

### 2.9. Statistical Analysis

The statistical analysis of the various experiments was performed by ANOVA and results confirmed by a Bonferroni *t*-test, with a *p* value of <0.05 considered statistically significant.

## 3. Results and Discussion

### 3.1. Physico-Chemical and Technological Characterization of Nanostructures

The initial step of the present work was focused on the physico-chemical characterization of GMO-nanostructures in the presence of different non-ionic stabilizers with the aim of developing innovative, potentially safe and stable colloidal formulations.

The multifunctional building units of the poloxamer family play an important role in the stabilization and preservation of the integrity of lyotropic liquid crystalline particles. Indeed, the length of the PPO and PEO chains, the molecular weights and the ratios between the hydrophilic and hydrophobic anchoring portions, known as the hydrophilic liquid balance (HLB), can really affect the extent of the polymer adsorption onto the surfaces of the nanosystems and thus influence their stability. As can be seen in Table 1, the nanosystems prepared with PL F-127 (97-100 PEO portions) showed an average size of 96 nm with a narrow size distribution, as compared to the nanosystems prepared with PL F-68 and PL 10500 (73 and 37 units, respectively), which evidenced a significant increase in their hydrodynamic diameter up to 229 and 327 nm, respectively. These data are in agreement with Chong et al., who demonstrated that a greater poly (ethylene oxide) chain length (as in the case of PL F-127) is related to higher HLB values of the polymer, and thus to significant stabilization of the nanostructures against flocculation. In addition, the greater molecular weight of PL F-127(~12,500 Da) as compared to PL F-68 (~8500 Da) and PL 10500 (~6500 Da) could be a potential contributing factor towards the stabilization efficacy of the co-polymer to form homogeneous, monodispersed nanostructures [38].

Another class of surfactants used in this investigation for the preparation of GMO- nanostructures belongs to the polysorbate family, which has structural characteristics comparable to those of the Pluronic family. As can be seen in Table 1, the formulations prepared in the presence of Tweens showed an average diameter of less than 200 nm and a polydispersity index inferior to 0.3, both suitable for possible systemic administration. The zeta potential of all samples showed negative values of more or less −20 mV, possibly due to the presence of trace amounts of free fatty acid in the GMO. In fact, the negative charge on the surface of nanostructures was mainly due to the ionization of the carboxylic residue of the oleic acid.

**Figure 1:**
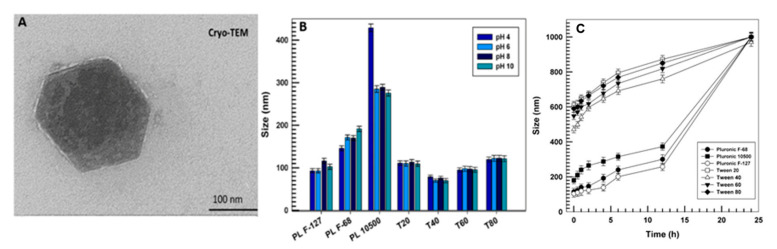
Cryo-TEM micrograph of GMO-nanostructures prepared with Pluronic F127 (PL F-127) (**A**); influence of pH on the mean diameter of nanostructures prepared with different non-ionic surfactants (**B**); serum stability of nanosystems prepared with different non-ionic surfactants in 70% fetal bovine serum (FBS) as a function of time (**C**).

As can be seen in Figure 1A, the nanostructures prepared with PL F-127 were hexagonal in shape with a thinner electrodense coating on the outer sides of the hexagon and a hydrodynamic diameter of about 100 nm, confirming the data obtained through dynamic light scattering (DLS) (micrographs of the other formulations are not shown because they were similar to the reported micrograph).

The stability of the nanostructures was also investigated. This was done by monitoring the mean size of the various nanosystems as a function of different pH values. As can be seen in Figure 1B, all the samples maintained an average diameter of less than 200 nm at all investigated pHs, with the exception of the sample containing PL 10500. This sample evidenced a dramatic increase in diameter (greater than 400 nm), which was probably due to its shorter hydrophilic chain. This may have reduced the adsorption and incorporation of the polymer onto the surfaces of the nanostructures, thus promoting such pronounced flocculation.

The compatibility of GMO-nanostructures with biological fluids is another aspect that needs to be investigated before in vitro and in vivo experiments. For this reason, the nanosystems were incubated in a medium containing 70% FBS at 37 °C and their size was evaluated for up to 24 h (Figure 1C). It is interesting to observe that the use of Tweens as stabilizers caused an increase in the average size of the nanostructures after only a few hours and this trend was accentuated as a function of the incubation times. This was probably due to the peculiar ability of polysorbate derivatives to adsorb plasmatic protein (i.e., apolipoproteins E and B) onto the surfaces of the nanosystems, thus inducing the formation of a corona, which was able to increase their hydrodynamic ray [31]. Conversely, the formulations prepared with Pluronics retained their average size (<200 nm) for up to 12 h, confirming the great compatibility of nanostructures made up of poloxamers with biological fluids.

The stability of the nanostructures against the destabilizing events promoted by the heating process is another important aspect to be evaluated during the pre-formulation phases of a pharmaceutical [32]. As shown in Table 2, the nanosystems prepared with PL F-127 and the various Tweens were subjected to heating and produced homogeneous and monodispersed colloidal dispersions. In fact, the hydrodynamic diameter for these samples was less than 200 nm and the polydispersity index value was between 0.1–0.2, thus confirming that the addition of these surfactants during the preparation of the nanocarriers significantly improved their stability. On the other hand, the formulations prepared with PL F-68 and PL 10500 showed a substantial increase in their average size and size distribution, suggesting that the increase in temperature may cause considerable destabilization of the colloidal structure.

Lyophilization studies were also carried out as a function of different cryoprotectants such as glucose, saccharose, sucrose, mannitol and trehalose at different concentrations (5–10% *w*/*v*), in order to provide a practical application of the nanosystems and thus assure long-term storage and stability. The formulations prepared with PL F-127 provided the best results in terms of mean size and size distribution only with the aid of mannitol at 5% and 10% *w*/*v*, whereas it was possible to resuspend the nanosystems prepared with PL F-68 after the freeze-drying process by adding mannose at 5% and 10% *w*/*v* (Table S1). Contrarily, the PL 10500 nanostructures again showed a significant increase in the aforementioned parameters, suggesting the presence of macroaggregates upon rehydration (Table S1).

Moreover, the use of 10% mannitol and mannose showed a tolerable increase in the mean size and polydisperpersity index of most of the Tween-nanostructures, demonstrating that these formulations could be dehydrated by freeze-drying, offering a useful powdered formulation for diverse applications (Table S2).

### 3.2. Physico-Chemical Characterization of DOX-Loaded GMO-Nanostructures

Considering the previous data, the nanostructures prepared with PL F-127 and T80 were chosen as suitable nanocarriers for the encapsulation of a hydrophilic model compound, which was doxorubicin hydrochloride. The addition of the compound in the aqueous phase promoted a slight increase in the mean diameter and size distribution of both nanosystems, demonstrating the absence of any significant destabilization of the colloidal formulations (Table 3).

Contrarily, the surface charge of the nanosystems was not strongly affected by the anticancer drug, showing negative values similar to those of the empty formulations. This aspect could be useful for potential intravenous injection because the presence of macroaggregates would be excluded [39]. However, aggregates did result when more than 0.6 mg/mL of DOX was initially used, as was demonstrated by the high values of the polydispersity index (Table 3), so it will not be considered for further investigation.

**Figure 2:**
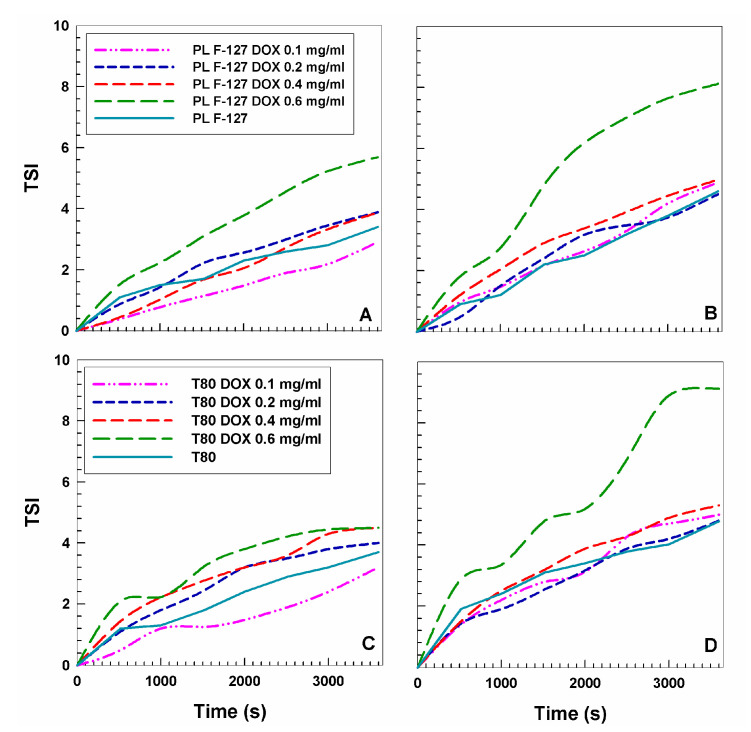
Turbiscan stability index (TSI) of GMO-nanostructures prepared with various amounts of DOX as a function of time and temperature (20 °C: **A**,**C**; 37 °C: **B**,**D**).

The stability of the nanostructures containing the anticancer drug at room temperature (20 °C) and at body temperature (37 °C) was also investigated by means of a Turbiscan Lab^®^ Expert apparatus, which is able to correlate the transmittance and backscattering of a formulation as a function of time and temperature [40,41]. The resulting data confirmed that the encapsulation of the active compound did not induce any significant variation in the TSI profiles at room temperature in both nanosystems as compared to the empty formulations, suggesting that the nanostructures effectively retain the drug with no destabilizing phenomena across time (Figure 2).

On the contrary, the formulation containing 0.6 mg/mL of the active compound showed a considerable increase in the kinetic profile values at 37 °C in both nanostructures, providing evidence of the destabilization induced by the greater amount of drug on the colloidal structure (Figure 2).

#### Evaluation of the Entrapment Efficiency and Release Profiles of DOX

**Figure 3:**
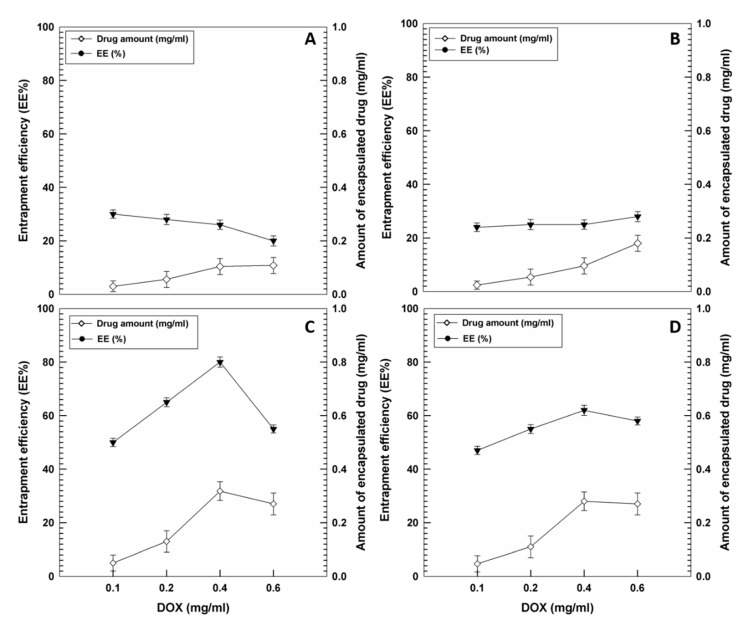
Entrapment efficiency (EE%) of DOX in GMO-nanostructures prepared with PL F-127 (**A**,**C**) and Tween 80 (T80) (**B**,**D**) before (**A**,**B**) and after pre-treatment (**C**,**D**) with ammonium sulfate as a function of the drug concentration initially used. Values represent the mean of three different experiments ± standard deviation.

The entrapment efficiency of the bioactive was investigated in order to obtain useful information on the capacity of the different nanostructures to retain the drug for in vivo applications [42]. In detail, various amounts of DOX were added to the aqueous phase during the sample preparation and in Figure 3A,B it is possible to observe the very slight degree of encapsulation as compared to the amount of drug initially added. In particular, the use of 0.1 and 0.2 mg/mL of DOX favored an encapsulation of about ~30% of the active compound in the PL F-127 and T80 nanosystems, respectively. A lesser entrapment efficiency of DOX resulted when 0.4 or 0.6 mg/mL of active compound were initially used (<30%), promoting a slight increase in the drug retention in the systems. The low degree of encapsulation of the drug was probably due to the rapid leakage of the compound from the bilayer resulting in its reduced retention in the nanosystems. Increasing amounts of DOX (>0.6 mg/mL) added during the preparation procedure of the GMO-nanostructures promoted a destabilization of the colloidal systems, producing sediments and macroaggregates (data not shown).

One useful approach for improving the entrapment efficiency of DOX is the remote loading method, which is normally proposed for doxorubicin-loaded liposomes such as Doxil^®^, the first FDA approved nanodrug. In detail, the pretreatment of liposomes with ammonium sulfate causes the protonation of amino groups of doxorubicin and thus the formation of a gel-like precipitate, which improves the retention of the drug inside the vesicles. The positively charged DOX is no longer able to cross the bilayer and remains efficiently entrapped, thus resulting in a successfully high dosage of the anticancer drug in the liposomal formulations. For this purpose, pre-treatment with ammonium sulfate was performed in the GMO-nanostructures prepared with PL F-127 and T80 in order to successfully improve the degree of drug encapsulation.

As shown in Figure 3C, the best results were obtained using 0.4 mg/mL of drug and PL F-127 as stabilizer because ~80% of the DOX was retained by the GMO-nanostructures, representing the best condition in terms of the cost/benefit ratio. It was interesting to observe a substantial decrease in entrapment efficiency (55%) when the amount of drug initially added was 0.6 mg/mL. This phenomenon suggests that a saturation of the inner compartments of the systems occurs, which makes them incapable of holding any more drugs. The same trend was obtained using T80 as surfactant, although a slighter encapsulation of the drug was achieved as compared to the systems prepared with PL F-127 (Figure 3D).

**Figure 4:**
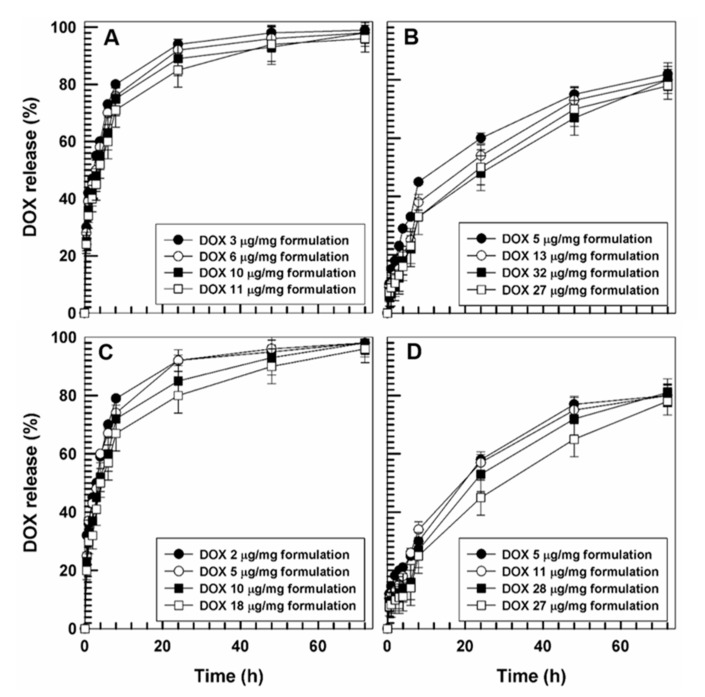
Release profile of DOX from GMO-nanostructures (μg/mg) prepared with PL F-127 (**A**,**B**) or T80 (**C**,**D**) before (**A**,**C**) and after (**B**,**D**) pre-treatment with ammonium sulfate as a function of the entrapped drug and incubation time. Values represent the mean of three different experiments ± standard deviation.

The next step focused on the evaluation of the drug release profiles from the GMO-nanostructures, before and after using the remote loading method. It was interesting to observe (Figure 4 A,C) that a great amount of DOX is released from nanostructures prepared in the absence of ammonium sulfate after a few hours incubation, confirming the results of encapsulation efficiency previously reported [25].

On the other hand, the evaluation of DOX leakage from the nanosystems through the use of a remote loading method showed a different kinetic profile. As shown in Figure 4B, the drug leakage from the GMO-nanostructures prepared with PL F-127 was constant and prolonged over time and was modulated by the amount of the drug. In fact, the samples containing lower amounts of drug (0.1 and 0.2 mg/mL) were characterized by a release of ~85% after 72 h, while the formulation containing the greatest amount of DOX was characterized by a leakage of ~70% after three days of analysis (Figure 4B).

The same trend was observed when the GMO-nanostructures were prepared using T80, confirming an increased retention of doxorubicin hydrochloride within the vesicles, subsequent to pretreatment with ammonium sulphate (Figure 4D).

### 3.3. Cytotoxicity of GMO-Nanostructures

**Figure 5:**
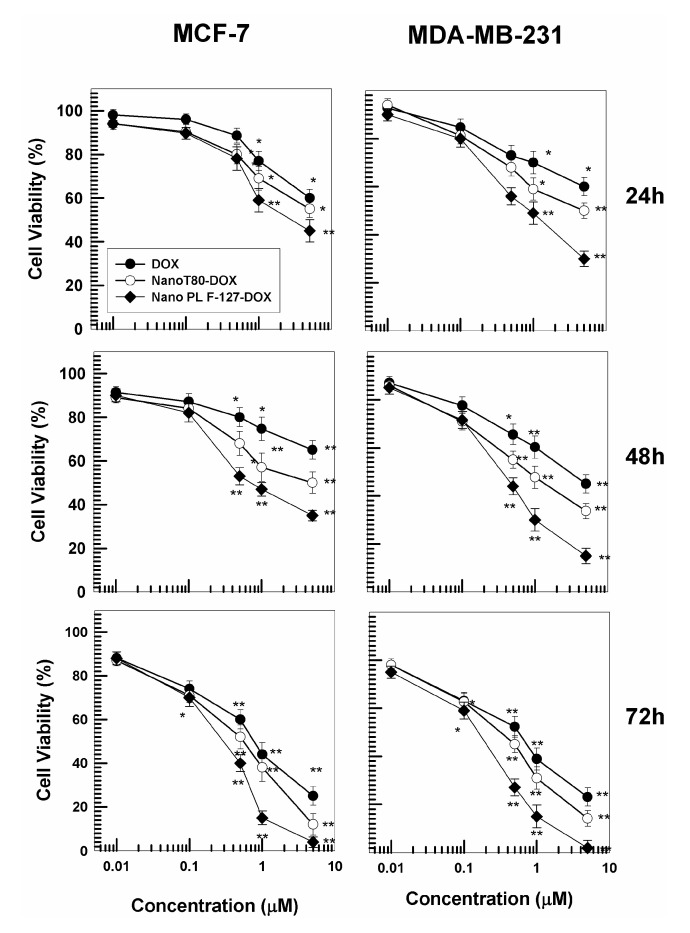
Evaluation of in vitro cytotoxicity of GMO-nanostructures containing DOX prepared using 0.4 mg/mL of active compound (32 μg of drug/mg of formulation prepared with PL F-127 and 27 μg of drug/mg of formulation prepared with T80 ) on MCF-7 and MDA-MB-231 cells as a function of the drug concentration and incubation time. Data are the percentages of cellular viability as evaluated by MTT-testing. Results are the mean of four different experiments ± standard deviation. * *p* < 0.05, ** *p* < 0.001 (with respect to the untreated cells).

The cytotoxicity of nanostructures is another aspect that needs to be investigated in the phases of preformulation [43]. For this reason, the systems prepared with both stabilizers containing 0.4 mg/mL of DOX were chosen to evaluate the pharmacological efficacy of the nanosystems. In detail, the cytotoxicity of the nanostructures was investigated on MCF-7 and MDA-MB-231 cells, as models of human cancer cells sensitive to the hydrophilic drug, with respect to the free form of the bioactive as a function of all the incubation times (24, 48, or 72 h) and the drug concentrations (0.1–5 μM) (Figure 5). The nanoencapsulation of DOX in both nanostructures promoted a greater decrease in cell viability as compared to the free form of the drug at all the concentrations used (Figure 5). The best pharmacological activity was exerted by DOX-loaded PL F-127 nanostructures, especially on the MDA-MB-231 cells, probably because this cell line is slightly more sensitive to the hydrophilic drug (Figure 5). Indeed, the formulation prepared with poloxamer induced an increased cytotoxicity of doxorubicin hydrochloride after just 24 h and at a drug concentration of 0.5 μM.

In order to further confirm the pharmacological activity of the DOX-loaded GMO nanostructures on breast cancer cell lines, the trypan blue dye exclusion test was performed [44].

**Figure 6:**
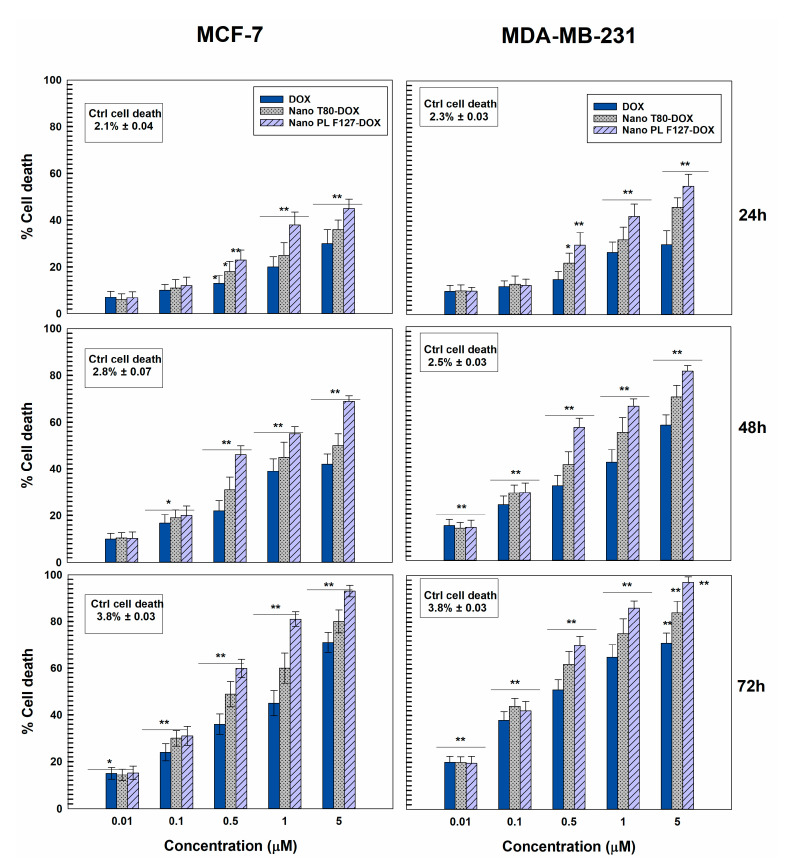
Mortality rates in MCF-7 and MDA-MB-231 cells treated for 24, 48, or 72 h with different concentrations of DOX free or entrapped in GMO-nanostructures prepared with PL F-127 and T80. Results are the mean of four different experiments ± standard deviation, expressed as percentage of death cells over total counted. * *p* < 0.05, ** *p* < 0.001 (with respect to the untreated cells).

Treatment of MCF-7 and particularly MDA-MB-231 cells with GMO-nanosystems resulted in significant cell death (Figure 6) when compared to the control and free form of DOX.

In detail, the nanostructures prepared with both stabilizers containing DOX promoted a significant increase in cell death early on during incubation on MCF-7 and MDA-MB-231 cells, but once more, the best results in terms of pharmacological activity were exerted by the nanostructures prepared with PL F-127. This trend became even more evident in both cell lines after 48 h and is probably related to the ability of poloxamers to counteract the phenomenon known as multidrug resistance (MDR), which eventually leads to the failure of a plethora of promising anticancer agents.

Several works describe the capacity of the poloxamer family to sensitize the multidrug-resistant (MDR) tumor cells by inhibiting the cell membrane’s drug-efflux transporters of the ATP-binding cassette (ABC) superfamily, such as P-glycoprotein (Pgp), multidrug resistance proteins (MRPs) and breast cancer resistance proteins (BCRPs) [45,46], causing a considerable reduction in ATP levels in cells [15]. Several small molecular MDR inhibitors such as verapamil or cyclosporine A have been used to overcome the increased drug efflux, but their lack of specificity together with their intrinsic toxicity limits their clinical application [47].

**Figure 7:**
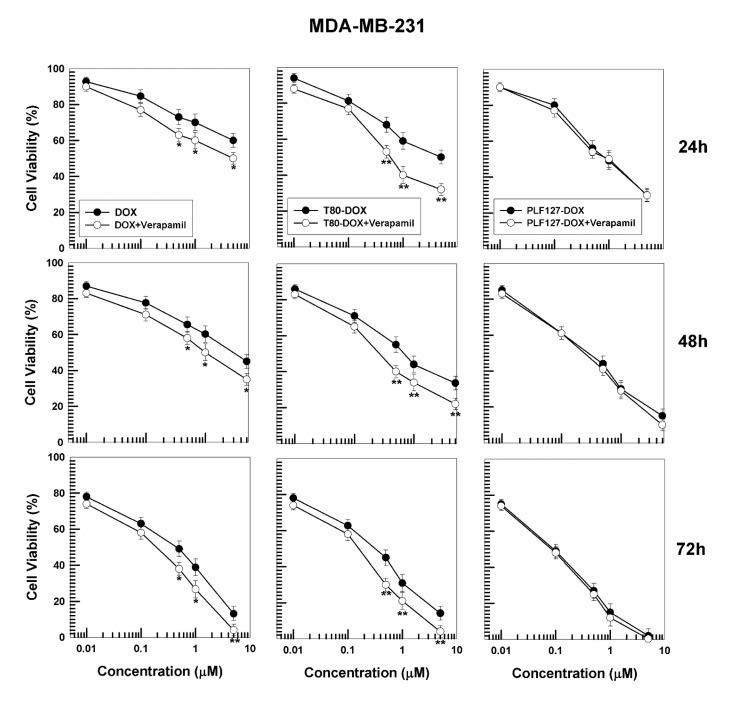
Evaluation of in vitro cytotoxicity of GMO-nanostructures containing DOX (prepared using 0.4 mg/mL of active compound (32 μg of drug/mg of formulation prepared with PL F-127 and 27 μg of drug/mg of formulation prepared with T80) on MDA-MB-231 cells, after pre-treatment with verapamil, as a function of the drug concentration and incubation time. Data are the percentages of cellular viability as evaluated by MTT-testing. Results are the mean of four different experiments ± standard deviation. * *p* < 0.05, ** *p* < 0.001 (with respect to the cells treated with DOX free and T80-DOX, respectively).

For this purpose, in order to validate the cytotoxic activity of the GMO-nanostructures containing poloxamer as stabilizer with respect to those prepared with polysorbate, the cytotoxicity of DOX-loaded nanostructures was investigated following pretreatment with verapamil. Namely, both cancer cell lines were incubated with verapamil and subsequently treated with the various formulations of DOX (Figure 7).

It is interesting to observe that verapamil undeniably improves the pharmacological activity of DOX and DOX-loaded T80 nanostructures on both cancer cell lines, while the nanostructures prepared with PL F-127 showed cytotoxic profiles similar to those of verapamil, confirming once again that this polymer acts as a strong P-gp inhibitor (Figure 7 and Figure S1). These data are in agreement with the study performed by Alvarez-Lorenzo et al., which highlights the dependence of the anti-P-gp activity of the poloxamers as a function of the PPO block, HLB, and low concentration of surfactant (<1%) [48]. The evaluation of the IC_50_ of the various DOX formulations confirmed the increased antitumor effect promoted by the nanoencapsulation of the active compound with respect to its free form (Table 4).

Such results are highly encouraging and suggest a positive therapeutic scenario that could bypass drug resistance in the treatment of breast cancer.

Based on the previous data, the nanostructures prepared using PL F-127 were chosen for further investigation concerning the rate of interaction between cells and colloidal systems through the use of radiolabelled markers (such as tritiated hexadecyl-cholesterol) added to the organic phase during the preparation of the formulations.

**Figure 8:**
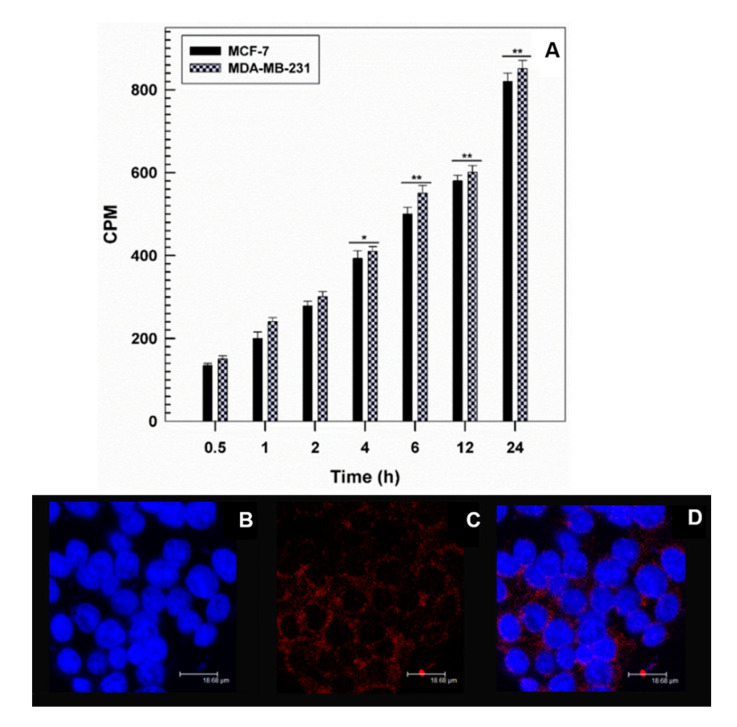
Interaction between [^3^H] cholesteryl hexadecyl ether (CHE) radiolabeled GMO-nanostructures and the different cancer cell lines as a function of incubation times (**A**). Results are the mean of three different experiments ± standard deviation. * *p* < 0.05, ** *p* < 0.001. Confocal laser microscopy (CLSM) micrographs of MDA-MB 231 cells incubated with rhodamine-1,2-dihexadecanoyl-*sn*-glycero-3-phosphoethanolamine triethylammonium salt (rhodamine-DHPE)-labeled GMO-nanosystems for 3 h. (**B**) Hoechst filter; (**C**) TRITC filter; (**D**) overlay. No auto-fluorescent phenomena were observed. Scale bar is 18.68 μm.

In detail, Figure 8A shows time-dependent cellular uptake of the nanosystems occurring after just 1 h incubation, followed by a huge uptake of the GMO-nanostructures after 24 h incubation. These results were confirmed by CLSM; in fact, significant interaction between the GMO-nanostructures containing rhodamine-DHPE and the cells came about after only a few hours, demonstrating the ability of these colloidal systems to enhance the cellular uptake of the entrapped drug (Figure 8B–D).

## 4. Conclusions

The peculiar features of nanostructures prepared with PL F-127 highlight the great potential of these systems to encapsulate and modulate the release of doxorubicin hydrochloride. When the remote loading approach is used to improve the efficiency of the entrapment of the bioactive by the nanostructures, a high retention rate of DOX is promoted in the vesicles, favoring a constant and prolonged drug release over 72 h.

Moreover, the nanoencapsulation of the anticancer agent in the GMO-nanostructures prepared with PL F127 showed a greater decrease in cell viability on MCF-7 and MDA-MB-231 cell lines with respect to the free form of DOX at all the drug concentrations tested.

Even though the values of the drug encapsulation are slightly lower than other systems such as Doxil^®^, the advantage of the proposed nanostructures is related to the use of inexpensive raw materials and to a simple preparation process as well as the presence of a low concentration of GMO and PL F-127 as stabilizers.

Indeed, the nanoformulation prepared with poloxamer as a stabilizer was shown to be potentially involved in the inhibition of the efflux pumps, although additional investigations should be performed in order to fully describe these mechanisms, which can dramatically improve the pharmacological output of DOX-loaded GMO-nanostructures in anticancer therapy.

However, additional studies concerning the investigation of the antitumor properties of DOX-loaded PL F127-GMO nanostructures on murine tumor xenograft models are in progress in order to evaluate the actual efficacy of this nanomedicine in preclinical and clinical applications.

## Figures and Tables

**Table 1:** Physico-chemical properties of glyceryl monooleate (GMO)-nanostructures.

Sample	Molecular Weight	HLB	Mean Sizes (nm)	Polydispersity Index	Zeta Potential (mV)
PL F-127 ^a^	12,500	18–23	96 ± 2	0.11 ± 0.02	−27 ± 1
PL F-68 ^b^	8500	29	229 ± 1	0.19 ± 0.03	−17 ± 2
PL 10500 ^c^	6500	15	327 ± 2	0.34 ± 0.05	−20 ± 2
T20 ^d^	1227	16.7	108 ± 1	0.09 ± 0.01	−27 ± 1
T40 ^e^	1284	15.6	105 ± 2	0.31 ± 0.02	−29 ± 1
T60 ^f^	1311	14.9	93 ± 1	0.11 ± 0.03	−26 ± 2
T80 ^g^	1310	15	120 ± 2	0.07 ± 0.05	−26 ± 2

^a^ Nanostructures prepared with Pluronic F-127; ^b^ nanostructures prepared with Pluronic F-68; ^c^ nanostructures prepared with Pluronic 10500; ^d^ nanostructures prepared with Tween 20; ^e^ nanostructures prepared with Tween 40; ^f^ nanostructures prepared with Tween 60; ^g^ nanostructures prepared with Tween 80.

**Table 2:** Effect of heating on the physical-chemical parameters of the various GMO-nanostructures.

Sample	Temperature (°C)	Mean Size (nm)	Polydispersity Index	Zeta Potential (mV)
PL F127	Before heating	96 ± 2	0.11 ± 0.02	−27 ± 1
	30	114 ± 6	0.28 ± 0.02	−22 ± 1
	40	114 ± 6	0.28 ± 0.07	−21 ± 1
	50	118 ± 6	0.31 ± 0.08	−20 ± 2
PL F68	Before heating	229 ± 1	0.19 ± 0.03	−17 ± 2
	30	220 ± 8	0.32 ± 0.01	−23 ± 1
	40	224 ± 8	0.35 ± 0.01	−23 ± 1
	50	242 ± 8	0.31 ± 0.01	−22 ± 1
PL 10500	Before heating	327 ± 2	0.34 ± 0.05	−20 ± 2
	30	381 ± 9	0.32 ± 0.03	−22 ± 1
	40	354 ± 9	0.33 ± 0.02	−23 ± 1
	50	389 ± 9	0.36 ± 0.01	−23 ± 1
T20	Before heating	108 ± 1	0.09 ± 0.01	−27 ± 1
	30	112 ± 5	0.16 ± 0.01	−26 ± 2
	40	120 ± 6	0.26 ± 0.02	−25 ± 2
	50	115 ± 5	0.11 ± 0.02	−24 ± 1
T40	Before heating	105 ± 2	0.31 ± 0.02	−29 ± 1
	30	72 ± 3	0.24 ± 0.01	−30 ± 3
	40	76 ± 4	0.20 ± 0.02	−25 ± 2
	50	72 ± 4	0.20 ± 0.02	−26 ± 1
T60	Before heating	93 ± 1	0.11 ± 0.03	−26 ± 2
	30	96 ± 1	0.10 ± 0.01	−26 ± 2
	40	96 ± 2	0.11 ± 0.02	−20 ± 2
	50	101 ± 1	0.10 ± 0.02	−20 ± 1
T80	Before heating	120 ± 2	0.07 ± 0.05	−26 ± 2
	30	126 ± 6	0.13 ± 0.01	−27 ± 3
	40	132 ± 7	0.22 ± 0.02	−28 ± 2
	50	128 ± 6	0.13 ± 0.02	−26 ± 1

**Table 3:** Physico-chemical properties of doxorubicin hydrochloride (DOX)-loaded GMO-nanostructures.

Nano F-127			
DOX Concentration (mg/mL)	Mean Sizes (nm)	Polydispersity Index	Zeta Potential (mV)
0.1	94 ± 1	0.19 ± 0.02	−17 ± 1
0.2	97 ± 5	0.14 ± 0.04	−20 ± 1
0.4	112 ± 6	0.18 ± 0.08	−22 ± 2
0.6	122 ± 7	0.22 ± 0.04	−22 ± 2
0.8	280 ± 5	0.47 ± 0.02	−16 ± 2
**Nano T80**			
0.1	120 ± 1	0.12 ± 0.02	−24 ± 1
0.2	132 ± 4	0.19 ± 0.04	−22 ± 1
0.4	150 ± 6	0.21 ± 0.08	−25 ± 2
0.6	185 ± 7	0.28 ± 0.04	−27 ± 2
0.8	296 ± 5	0.5 ± 0.06	−20 ± 2

**Table 4:** IC_50_ value of DOX and DOX-loaded GMO nanostructures as a function of cell lines and incubation time.

Cell Lines	Incubation Time	IC_50_ DOX	IC_50_ T80 DOX	IC_50_ PL F127 DOX
MCF-7	24 h	10.16	8.71	1.71
	48 h	9.79	5.18	0.66
	72 h	0.75	0.52	0.28
MDA-MB-231	24 h	9.41	3.72	0.86
	48 h	2.86	0.79	0.32
	72 h	0.46	0.29	0.15
**Cell lines**	**Incubation time**	**IC_50_ DOX+Verapamil**	**IC_50_ T80-DOX+Verapamil**	**IC_50_ PLF127-DOX+Verapamil**
MCF-7	24 h	1.65	1.21	1.76
	48 h	1.29	0.67	0.30
	72 h	0.29	0.23	0.19
MDA-MB-231	24 h	4.3	0.86	0.55
	48 h	1.1	0.21	0.21
	72 h	0.21	0.11	0.19

## Supplementary Materials

The following are available online at https://www.mdpi.com/1999-4923/12/11/1017/s1, Table S1: Physico-chemical properties of GMO-nanostructures after the freeze-drying process as a function of surfactant and cryoprotectant used, Table S2: Physico-chemical properties of GMO-nanostructures after the freeze-drying process as a function of surfactant and cryoprotectant used. Figure S1: Evaluation of in vitro cytotoxicity of GMO-nanostructures containing DOX (prepared using 0.4 mg/mL of active compound) on MCF-7 cells, after pre-treatment with verapamil, as a function of the drug concentration and incubation time.
