# Supplementary material for: Design and Characterization of Glyceryl Monooleate-Nanostructures Containing Doxorubicin Hydrochloride
Source: Pharmaceutics. 2020 Oct 24;12(11):1017. doi: 10.3390/pharmaceutics12111017 (PMC7690907; doi:10.3390/pharmaceutics12111017)
Supplement: Supplementary file 1 [file pharmaceutics-12-01017-s001.pdf]

pharmaceutics

Supplementary Materials: Design and Characterization of Glyceryl Monooleate-Nanostructures Containing Doxorubicin Hydrochloride

Agnese Gagliardi, Donato Cosco, Betty P. Udongo, Luciana Dini, Giuseppe Viglietto and Donatella Paolino

Table S1. Physico-chemical properties of GMO-nanostructures after the freeze-drying process as a function of surfactant and cryoprotectant used.

Sample	Cryoprotectant (% w/v)	Mean size (nm)	Polidispersity Index	Zeta potential (mV)
PL F-127	Glucose 5%	380±12	0.4±0.01	-32±1
	Glucose 10%	> 1000	0.3±0.11	-33±1
	Trehalose 5%	325±16	0.4±0.05	-34±1
	Trehalose 10%	340±15	0.4±0.02	-32±1
	Mannose 5%	319±14	0.4±0.12	-29±1
	Mannose 10 %	533±21	0.8±0.09	-29±2
	Sucrose 5%	332±14	0.5±0.06	-11±1
	Sucrose 10%	483±18	0.4±0.01	-24±1
	Mannitol 5%	253±8	0.2±0.05	-27±1
	Mannitol 10%	194±9	0.2±0.06	-34±1
PL F-68	Glucose 5%	580±15	0.8±0.01	-24±3
	Glucose 10%	> 1000	0.3±0.31	-28±2
	Trehalose 5%	433±16	0.5±0.05	-34±1
	Trehalose 10%	306±15	0.4±0.02	-30±2
	Mannose 5%	260±14	0.2±0.12	-26±4
	Mannose 10 %	252±21	0.2±0.09	-29±3
	Sucrose 5%	320±14	0.2±0.06	-11±1
	Sucrose 10%	251±18	0.4±0.01	-29±3
	Mannitol 5%	306±11	0.3±0.05	-16±1
	Mannitol 10%	294±8	0.8±0.06	-20±1
PLX188	Glucose 5%	> 1000	0.9±0.17	-24±1
	Glucose 10%	379±7	0.3±0.28	-25±1
	Trehalose 5%	521±32	0.5±0.05	-28±1
	Trehalose 10%	484±30	0.6±0.06	-25±2
	Mannose 5%	> 1000	0.4±0.01	-24±3
	Mannose 10 %	> 1000	0.9±0.17	-22±5
	Sucrose 5%	299±3	0.8±0.27	-30±1
	Sucrose 10%	253±6	0.7±0.21	-19±6
	Mannitol 5%	> 1000	0.9±0.06	-29±3
	Mannitol 10%	> 1000	0.8±0.01	-17±5

Table S2. Physico-chemical properties of GMO-nanostructures after the freeze-drying process as a function of surfactant and cryoprotectant used.

Sample	Cryoprotectant (% w/v)	Mean size (nm)	Polidispersity Index	Zeta potential (mV)
**Tween 20**	Glucose 5%	238±3	0.7±0.03	-36±2
	Glucose 10%	>1000	0.9±0.04	-33±3
	Trehalose 5%	>1000	0.5±0.03	-37±1
	Trehalose 10%	>1000	0.7±0.02	-36±4
	Mannose 5%	454±3	0.7±0.04	-39±5
	Mannose 10 %	252±31	0.2±0.03	-29±2
	Sucrose 5%	138±2	0.5±0.27	-38±2
	Sucrose 10%	>1000	0.6±0.21	-34±3
	Mannitol 5%	>1000	0.6±0.05	-40±7
	Mannitol 10%	178±3	0.2±0.04	-29±2
**Tween 40**	Glucose 5%	>1000	0.9±0.01	-31±2
	Glucose 10%	>1000	0.6±0.11	-34±3
	Trehalose 5%	>1000	0.9±0.05	-32±2
	Trehalose 10%	>1000	0.9±0.02	-30±3
	Mannose 5%	319±3	0.4±0.12	-26±7
	Mannose 10 %	533±4	0.8±0.09	-29±2
	Sucrose 5%	>1000	0.9±0.06	-34±6
	Sucrose 10%	483±5	0.42±0.01	-29±1
	Mannitol 5%	253±11	0.5±0.05	-32±1
	Mannitol 10%	194±7	0.2±0.06	-38±2
**Tween 60**	Glucose 5%	535±7	0.7±0.17	-34±2
	Glucose 10%	369±7	0.6±0.28	-38±1
	Trehalose 5%	308±32	0.5±0.05	-35±2
	Trehalose 10%	>1000	0.9±0.06	-37±2
	Mannose 5%	>1000	0.4±0.01	-24±1
	Mannose 10 %	190±2	0.4±0.17	-33±3
	Sucrose 5%	334±2.9	0.4±0.27	-37±2
	Sucrose 10%	>1000	0.4±0.21	-32±2
	Mannitol 5%	>1000	0.4±0.06	-33±1
	Mannitol 10%	189±4	0.2±0.01	-39±2
**Tween 80**	Glucose 5%	>1000	0.5±0.12	-34±1
	Glucose 10%	488±32	0.9±1.22	-35±1
	Trehalose 5%	>1000	0.7±0.06	-37±2
	Trehalose 10%	803±22	0.6±0.19	-35±2
	Mannose 5%	200±3	0.3±0.23	-32±2
	Mannose 10 %	200±3	0.2±0.16	-30±3
	Sucrose 5%	430±7.9	0.7±0.24	-31±4
	Sucrose 10%	638±12	0.9±1.23	-38±6
	Mannitol 5%	>1000	0.9±0.19	-38±4.9
	Mannitol 10%	736±14	0.7±0.22	-29±4

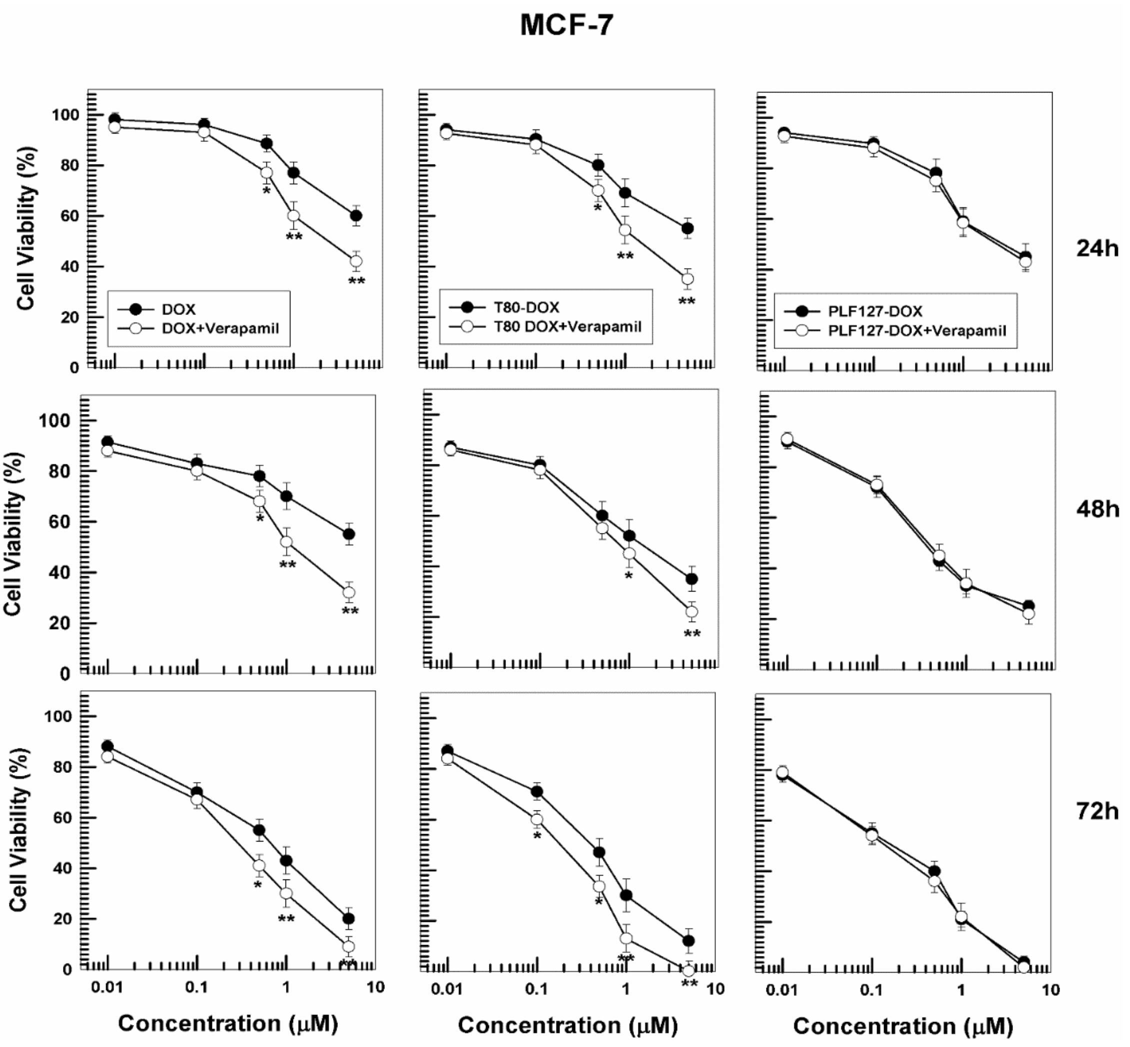

Figure S1. Evaluation of *in vitro* cytotoxicity of GMO-nanostructures containing DOX (prepared using 0.4 mg/ml of active compound) on MCF-7 cells, after pre-treatment with Verapamil, as a function of the drug concentration and incubation time. Data are the percentages of cellular viability as evaluated by MTT-testing. Results are the mean of four different experiments ± standard deviation. *$p < 0.05$, **$p < 0.001$ (with respect to the cells treated with DOX free and T80-DOX, respectively).
